# Strawberry agro-industrial by-products as a source of bioactive compounds: effect of cultivar on the phenolic profile and the antioxidant capacity

**DOI:** 10.1186/s40643-021-00416-z

**Published:** 2021-07-13

**Authors:** Esteban Villamil-Galindo, Franco Van de Velde, Andrea M. Piagentini

**Affiliations:** 1 Instituto de Tecnología de Alimentos, Facultad de Ingeniería Química, Universidad Nacional del Litoral, Santiago del Estero 2829, 3000 Santa Fe, Argentina; 2 Consejo Nacional de Investigaciones Científicas y Técnicas (CONICET), Santa Fe, Argentina

**Keywords:** Agro-industrial by-products, Strawberry cultivars, Revalorisation, Bioactive compounds, Hydrolysable tannins, Antioxidants

## Abstract

The post-harvest processing of strawberries generates considerable amounts of by-products that consist of the inedible parts of the fruit (sepal, calyx, stem, and non-marketable portion of the fruit), which is an environmental problem for local producers and industries. This study aimed to revalue these kinds of tissues through identifying and quantifying the genotype influence on the total phenolic content, phenolic profile, and the antioxidant activity of the by-products from three strawberry cultivars: ‘Festival’ (FE), ‘San Andreas ‘ (SA), and ‘Camino Real’ (CR). The total phenolic content was determined by the Folin–Ciocalteu method, in-vitro antioxidant activity by the DPPH* radical scavenging method and the phenolic profile by PAD–HPLC. The different genotypes showed significant differences (*p* < 0.05) in total phenolic content (TPC), FE being the one with the highest TPC (14.97 g of gallic acid equivalents < GAE > /Kg of by-product < R >), followed by SA and CR cultivars. The antioxidant capacity of the SA and FE tissues were similar (*p* > 0.05) and higher (15.1–16.3 mmol Trolox equivalents < TE > /Kg R) than CR. Eight main phenolic compounds were identified and quantified on the three cultivars. Agrimoniin was the principal polyphenol (0.38–1.56 g/Kg R), and the cultivar FE had the highest concentration. This compound showed the highest correlation coefficient with the antioxidant capacity (*R*^2^ 0.87; *p* < 0.001). This study highlighted the impact of the multi-cultivar systems in strawberry production on the bioactive potential and the diversity of secondary metabolites obtained from strawberry agro-industrial by-products at a low cost.
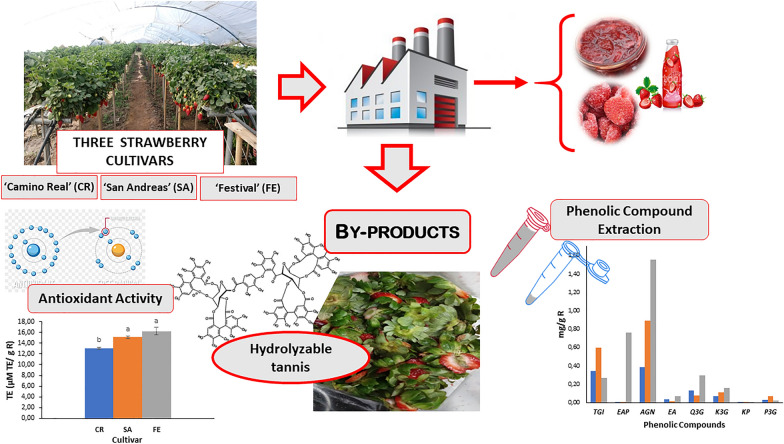

## Introduction

The agro-industrial processing of fruits and vegetables generates significant quantities of by-products. They consist of the inedible parts (peel, seeds, stems, leaves, etc.) or products outside of quality parameters. In many cases, these vegetable tissues are not properly disposed of, generating a severe impact on the environment (Girotto et al. 2015). Agro-industrial tissue wastes include many functional and nutraceutical compounds that can be recovered and used in many industries. They are fibre sources, mainly composed of lignocellulose (Ravindran et al. 2018), and also sources of numerous bioactive compounds such as vitamins, minerals, phenolic compounds, and terpenes, among others (Girotto et al. 2015).

The agroindustrial strawberry chain is one of the most important locally and worldwide. The strawberry belongs to the family of *Rosaceae* of the genus *Fragaria*, where we find the *Fragaria × ananassa* predominantly at the commercial level. Strawberry worldwide production is approximately 8,000,000 tons per year. In South America, Brazil, Argentina, and Chile are the principal producers with 320,000 tons per year with a dynamic growth (Antunes and Peres 2013). Argentina ranks 34th in world production, producing 50,000 tons, and ranks 17th in exports, the latter increasing exponentially. The increases are mainly due to improvements in crop production strategies, where the multi varietal systems and the micro and macro tunnels have improved yields and fruit quality (Axel 2016; Sordo et al. 2017).

‘Camarosa’ was one of the strawberry cultivars traditionally locally produced*.* Nevertheless, to improve yields, different cultivars have been used in Argentina, adapted to the diverse agro-ecological conditions. Examples are the short-day cultivars for the fall–winter–spring seasons, such as ‘Sabrosa’, ‘Ventana’, ‘Camino Real’, ‘Festival´, ‘Fortuna´, and ‘Splendor’. On the other hand, there are day-neutral cultivars for the fall–spring seasons, such as ‘Aromas’, ‘Albion’, and ‘San Andreas’ (Axel 2016)*.*

The wide availability of this fruit makes its industrial processing more rentable every day, through the production of IQF strawberries, jams, pulps, and juices, among others. At the same time, the amount of residue generated by this activity increases, representing up to 20% of the total production (Villamil-Galindo et al. 2020). These discarded plant tissues, which consist mainly of the sepals, stems, and part of the fruit remaining, can be proposed as a source of bioactive and functional compounds, since their composition includes metabolites of great interest as the phenolic compounds. According to Gündüz (2015), the different parts of the strawberry plant may show a different phenolic compound profile characterized by the presence of ellagitannins, flavonoids, and phenolic acids. For instance, ellagic acid was the main compound in the root and flower of the strawberry plant, followed by quercetin-3-*O*-glucoside (Zhu et al. 2015). The distribution of these metabolites is diverse and characteristic of each strawberry cultivar (Buendía et al. 2010; Van de Velde et al. 2019). In this regard, ‘Camarosa’ strawberries presented higher anthocyanin content than ‘Selva ‘ strawberries, while the latter exhibited higher ellagic acid content (Van De Velde et al. 2013). However, the variation on the phenolic compound profile and the antioxidant capacity of the strawberry by-products according to the cultivar they come from is not well known. These non-edible parts of the plant may have different quantities and content of phenolic compounds with great bioactive potential (Zhu et al. 2015).

Therefore, the objective of this work was to characterize and quantify the content of phenolic compounds and the antioxidant capacity of extracts obtained from the agro-industrial by-products of three strawberry cultivars: ‘Festival’, ‘Camino Real, and ‘San Andreas’, and to establish strategies for the recovery of these valuable compounds with potential use in the food, pharmaceutical, and chemical industry, from a low-cost raw material.

## Materials and methods

### Plant material

The by-products of strawberry (R) (*Fragaria × ananassa Duch)* cv ‘Festival’, ‘Camino Real’, and ‘San Andreas’ were obtained during the postharvest processing of strawberry, from one field at Coronda, Argentina (31°58′00″S 60°55′00″W). The by-products are constituted by sepals, peduncles, and non-marketable portion of the fruit.

The by-products of the three cultivars of strawberry were immediately transported to the Instituto de Tecnología de Alimentos-FIQ-UNL (Santa Fe Capital), weighed, packed in 40 µm polypropylene bags (sample aliquots of 300 g), and stored at − 18 °C. Before extraction assays, the samples were ground to a particle size of < 1 mm.

### Extraction procedure

Sequential extraction was performed with 2 stages with a final solid–liquid ratio of 1:10 with ratios of 1:5 in each stage (Villamil-Galindo et al. 2020). The extracting solution was 80% methanol with 0.5% formic acid. In each stage, the extract was homogenized with vortex and placed in an ultrasound bath (T40, Teslab, Buenos Aires, Argentina) for 15 min, and centrifuged at 12,000*g* for 20 min (Neofuge 18R Heal Force refrigerated centrifuge, Shanghai, China). The extraction process was performed in triplicate for each strawberry cultivar by-product.

### Analysis of phenolic compounds and antioxidant capacity

#### Total phenolic content

Total phenolic content (TPC) of the extracts was measured in triplicate by the Folin–Ciocalteu method, modified according to Rodríguez-Arzuaga et al. (2021). Gallic acid (Sigma-Aldrich, St. Louis, MO, USA) was used as the standard reagent (20–100 ppm). The TPC was expressed as g of gallic acid equivalents (GAE)/Kilogram of strawberry by-product (g GAE/Kg).

#### Phenolic compound profile

The polyphenolic profile was determined using an LC-20AT high-performance liquid chromatograph with a diode array detector and Lab Solutions for data processing and control software (Shimadzu Co., Kyoto, Japan). Separation was performed on a Gemini 5 µ C18 110 Å 250 × 4.6 mm hybrid reverse phase column attached to a guard column (Phenomenex Inc, CA, USA). Determinations were performed according to Villamil-Galindo et al. (2020). Identification of phenolic compounds was performed by comparing retention times and UV–Vis absorption spectra of standard phenolic compounds reported in strawberries and/or their by-products.

Quantification of the identified compounds was performed by the external standard method, using calibration curves of analytical standards (Sigma-Aldrich Inc.; St. Louis, MO, USA) of ellagic acid (EA) (0.03–0.5 mg/mL, *R*^2^ = 0.9979) and quercetin-3-*O*-glucoside (Q3G) (0.06–1.00 mg/mL, *R*^2^ 0.9979). Results were expressed as g of phenolic compound/Kg R.

#### Antioxidant capacity

Antioxidant capacity was performed using the DPPH* radical, according to the modified method proposed by Nowicka et al. (2019). The extract was stand to react with 200 µL of the methanolic solution (0.08 mM) of 2.2-diphenyl-1-picrylhydrazyl (DPPH*) (Sigma-Aldrich) for 2 h in the darkness. The reaction was performed by triplicate. Finally, the absorbance was measured at 515 nm in a microplate reader (Asus UVM 340, Cambridge, England). The reference reagent was 2,5,7,8-tetramethylchroman-2-carboxylic acid (Trolox) (Sigma-Aldrich). Antioxidant activity was expressed as mmol Trolox equivalents/gram of by-product (mmol TE/Kg R).

### Statistical analysis

All data obtained in this study were tested by analysis of variance (ANOVA) to determine the effect of cultivar on the content and profile of phenolic compounds and on the antioxidant capacity. Significant differences between means were determined by Tukey’s test at a 5% significance level. In addition, Pearson´s correlation test was applied to determine the correlation between the variables studied. The statistical analyses were performed through the STATGRAPHICS centurion XV software (StatPoint Technologies Inc., Warrenton, VA, USA).

## Results and discussion

### Total phenolic content and antioxidant capacity

**Fig. 1:**
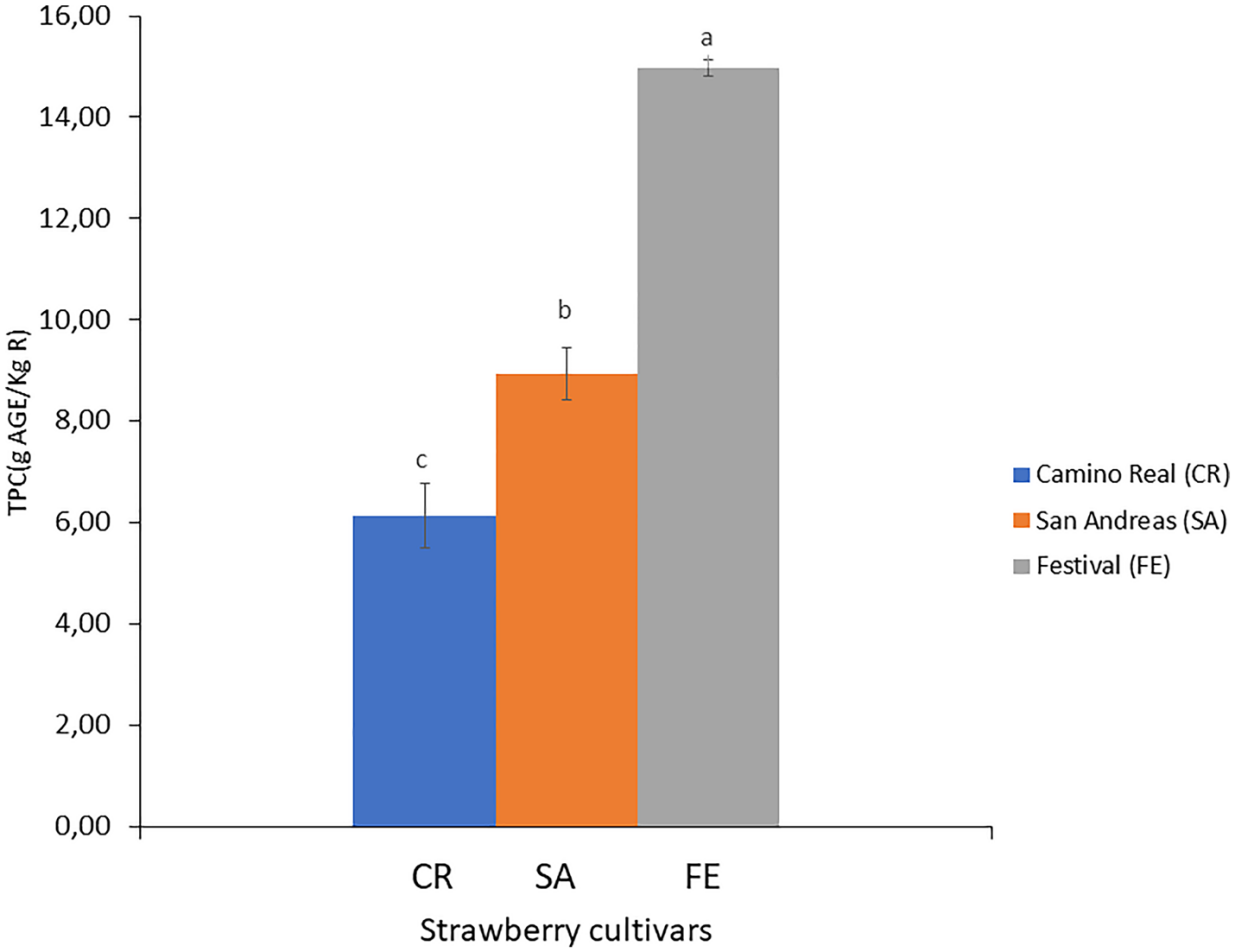
Total phenolic content (TPC) in the by-products of three strawberry cultivars. Different letters indicate significant differences (*p* < 0.05) among cultivars by Tukey’s test

The TPC of the by-products generated during the agro-industrial processing of three strawberry cultivars ‘Camino Real’ (CR), ‘San Andreas’ (SA), and ‘Festival’ (FE) was determined to evaluate their recovery, giving added value to this plant tissue as an alternative and low-cost source of bioactive compounds (Fig. 1).

The by-products from strawberry cultivar FE had the highest TPC (14.97 g GAE/Kg) among the three cultivars (*p* < 0.05), followed by the SA by-products (8.93 g GAE/Kg), and for the CR by-product (6.14 g GAE/Kg), with a TPC of about 42% of that determined for the FE cultivar (Fig. 1). The significant differences determined among the TPC of the residues of the three cultivars were probably due to intrinsic factors, such as the genotype of each plant (Di Vittori et al. 2018), and extrinsic factors, such as the production systems and the agro-ecological conditions. The ground concentration of some minerals (Zn, Fe, Mn, and Cu) is a key factor for the primary and secondary metabolism of plants, as well as the amount and type of the received radiation (Valentinuzzi et al. 2015). In Argentina, several strawberry production systems are managed, the seedlings can be exposed in open fields or under protection in macro-tunnels (Sordo et al. 2017). On the other hand, genotype also plays a fundamental role in the content of phenolic compounds and even more in plant tissues from the same field with similar growing conditions, as in this study. Connor et al. (2002) found a significant influence of genotype compared to the production environment and harvest year on the content of phenolic compounds and their antioxidant activity in different berries.

The highest TPC yield obtained for the by-products from strawberry cultivar FE could be related to the fact that, even though this cultivar is an early short-day cultivar and its exposure to solar radiation does not exceed 13 h to induce the flower bud, it is harvested on relatively cold days (4–8 °C), so it can be subjected to some abiotic stress due to low temperatures. Therefore, it may activate the phenylpropanoid metabolism in the tissues and favour the synthesis of phenolic compounds to protect itself from this adverse situation (Axel 2016; Gündüz 2015). On the contrary, the SA cultivar is an early day-neutral cultivar, with a flower bud is independent of the amount of time it is exposed to solar radiation, and harvested in the spring season with warmer temperatures close to 25 °C. This environmental condition would not elicit the activation of the phenylpropanoid metabolism (Axel 2016).

The TPC determined for the three strawberry cultivar by-products were lower than those reported by Simirgiotis et al. (2009) for Chilean wild strawberry sepals (20 g GAE/Kg) but higher than the phenolic-rich extracts obtained from blueberry leaves (8.59 g GAE/Kg), and green currant leaves (1.97 g GAE/Kg) (Fotirić Akšić et al. 2019; Raudsepp et al. 2010). Interestingly, the TPC obtained from the by-products of these three strawberry cultivars were higher than that determined in the own fruits. For the ‘Camino Real’ cultivar, Pineli et al. (2011) reported 2.1 g GAE/Kg strawberry, about one-third of that determined in this work in the by-product of the same cultivar (6.14 g GAE/Kg). In addition, higher phenolic contents were also obtained in the strawberry waste tissues analysed in this work than the total phenolic compounds reported for the fruit of other strawberry cultivars. For instance, the TPC of ‘Camarosa’ strawberry was 1.61 g GAE/Kg of fruit (Van de Velde et al. 2016), in order of the content of phenolic compounds reported by Nowicka et al. (2019) for other 90 strawberry cultivars at different maturity stages (0.08–2.08 g GAE/Kg fruit).

**Fig. 2:**
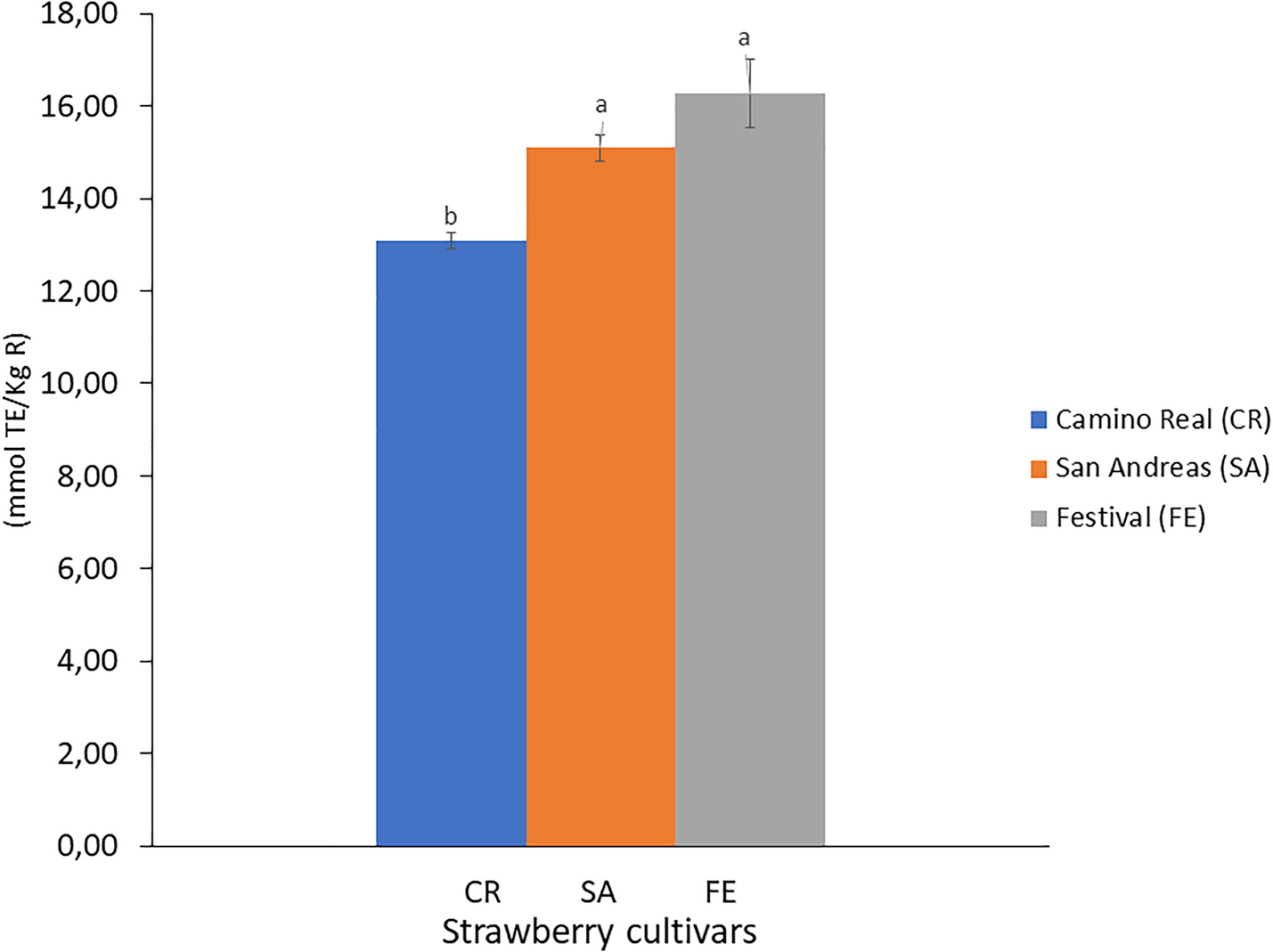
Antioxidant capacity of the by-products of three strawberry cultivars. Different letters indicate significant differences (*p* < 0.05) among cultivars by Tukey’s test

Regarding the antioxidant capacity, the agro-industrial FE and SA cultivar strawberry by-products presented similar values (*p* > 0.05) (16.3 and 15.1 mmol TE/Kg) (Fig. 2). These values, like those obtained for TPC, were higher than those reported for the fruits (strawberry) from ‘Alba’, ‘Irma’, and ‘Patty’ cultivars with approximately 13 mmol TE/Kg of fresh fruit (Tulipani et al. 2008). In turn, they are very similar to those determined for other agro-industrial by-products such as dogwood seeds with 19 mmol TE/Kg, a source of ellagitannins (Przybylska et al. 2020). It should be noted that the waste tissues of the three studied cultivars are very good sources of antioxidant compounds, with a great capacity to donate electrons or hydrogen, and thus neutralise different free radicals and other reactive oxygen species (Nowicka et al. 2019), although the strawberry by-product antioxidant capacity is cultivar dependant (*p* < 0.05). These extracts have an excellent potential for the nutraceutical industry. They are obtained from waste employing alternative green technologies such as ultrasound-assisted extraction with high industrial profitability, low environmental impact, and low cost (Vauchel et al. 2018).

### Phenolic compound profile

**Fig. 3:**
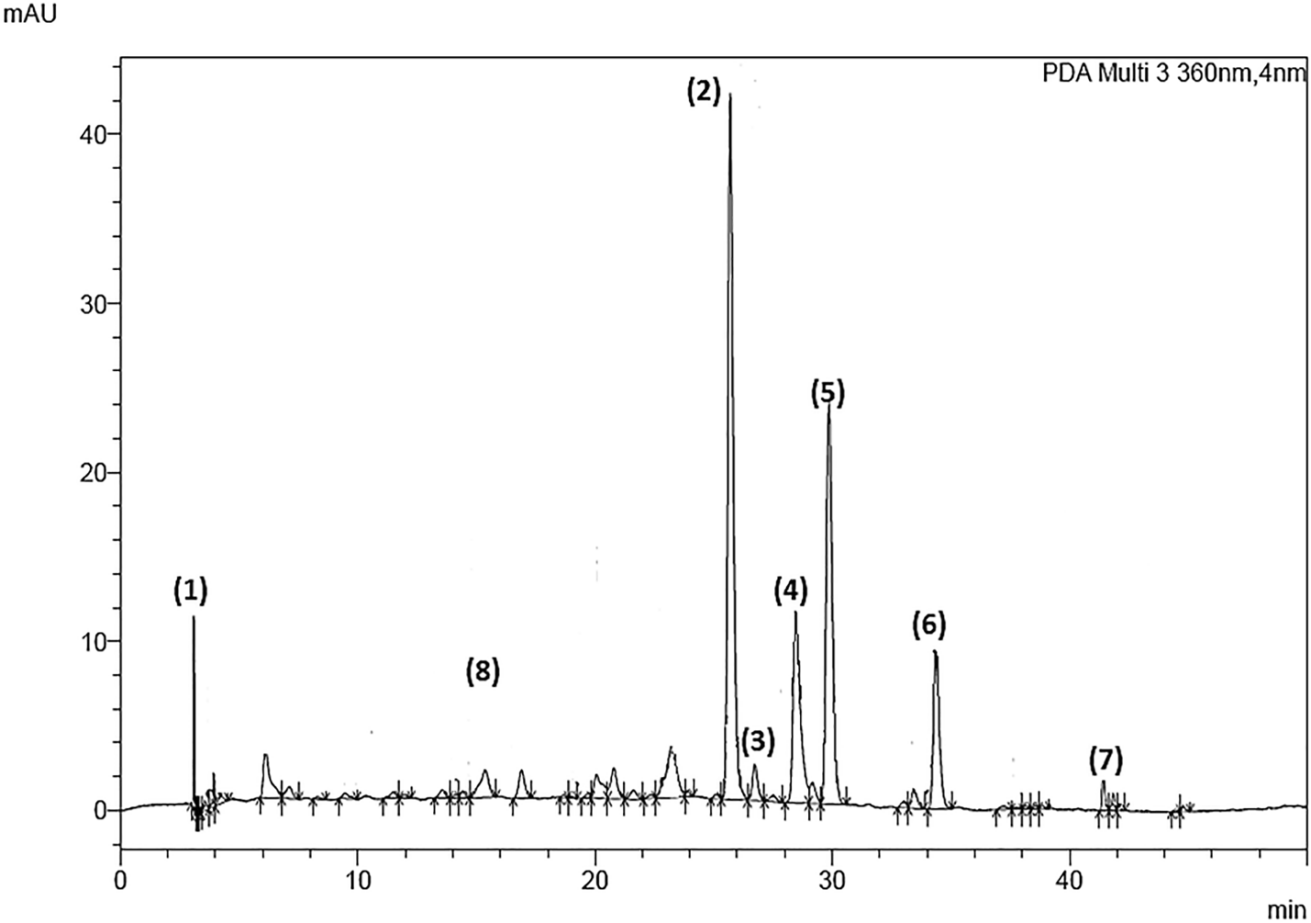
Typical chromatogram of polyphenol-rich extracts from the residues of three strawberry cultivars (360 nm wavelength). (1): tetragalloyl-glucose isomer, (2): ellagic acid pentoside, (3): agrimoniin, (4): free ellagic acid, (5): quercetin-3-*O*-glucuronide, (6): kaempferol-3-*O*-glucuronide, (7): kaempferol pentoside, (8): pelargonidin-3-*O*-glucoside

**Fig. 4:**
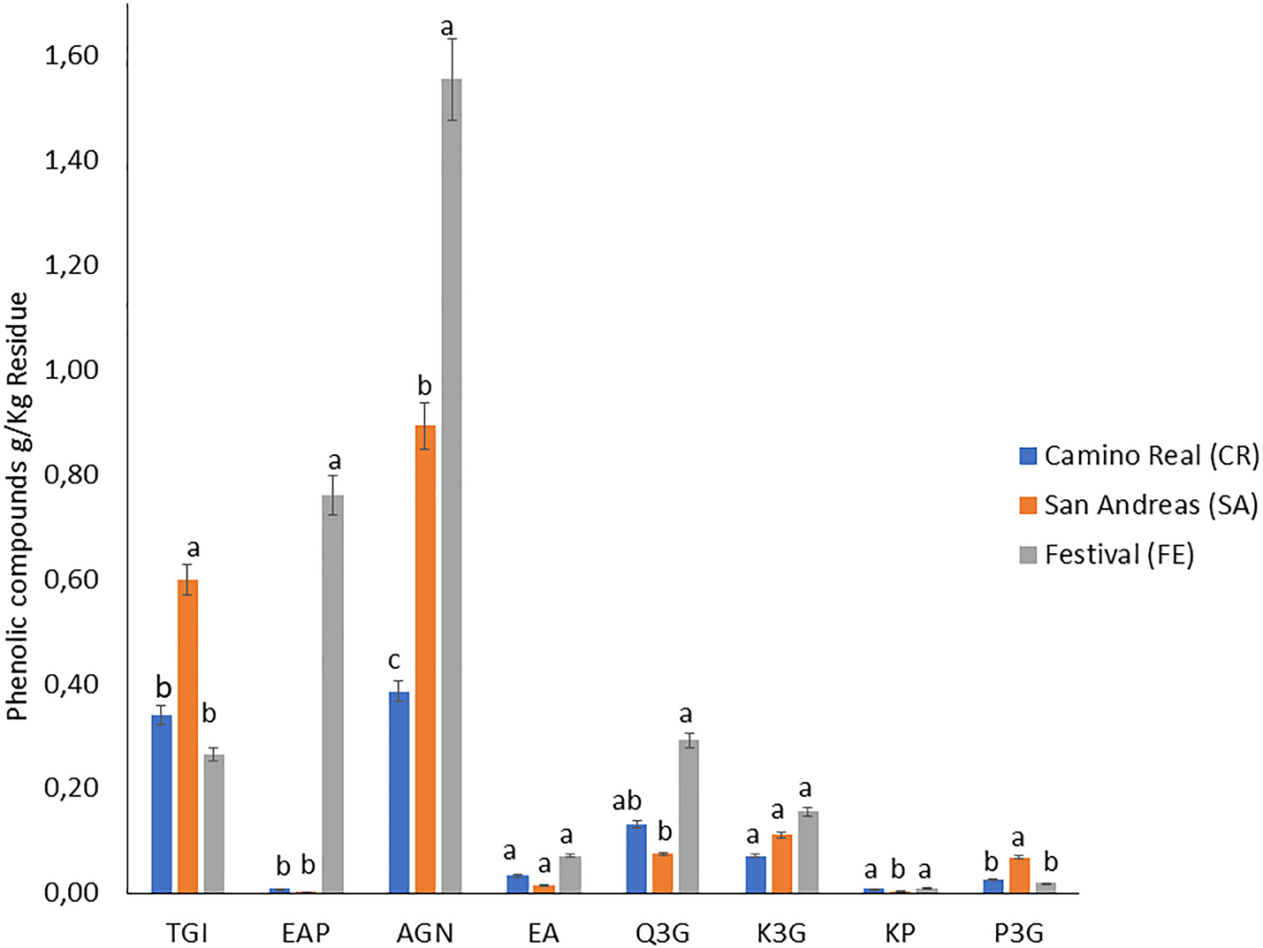
Quantification of phenolic compounds from strawberry by-products. *TGI* tetragalloyl-glucose isomer, *EAP* ellagic acid pentoside, *AGN* agrimoniin, *EA* free ellagic acid, *Q3G* quercetin-3-*O*-glucuronide, *K3G* kaempferol-3-*O*-glucuronide, *KP* kaempferol pentoside, *P3G* pelargonidin-3-*O-*glucoside. Different letters indicate significant differences (*p* < 0.05) among cultivars by Tukey’s test

Eight phenolic compounds were identified and quantified from the extracts of CR, SA, and FE cultivar strawberry by-products (Fig. 3). Hydrolysable tannins were the majority of the compounds determined in the three cultivars representing 83% in SA, 72% in CR, and 58% in FE, corresponding to tetragalloyl-glucose isomer and galloyl-bis-HHDP-glucose dimer (agrimoniin). The ellagic acid derivatives followed with 26% in FE, 4.1% in CR, and 2.5% in SA, represented by ellagic acid pentoside and free ellagic acid. Flavonols represented 21.2% in CR, 14.6% in FE, and 10.6% in SA, corresponding to quercetin-3-*O*-glucuronide, kaempferol-3-*O*-glucuronide, and kaempferol pentoside. Finally, anthocyanins represented 3.8% in SA, 2.6% in CR and 0.6% in FE, corresponding to pelargonidin-3-*O*-glucoside (Fig. 4).

According to the results, the distribution and concentration of phenolic compounds varied significantly (*p* < 0.05) among the waste tissues of the three strawberry cultivars studied.

The highest concentration (*p* < 0.05) of the tannin tetragalloyl-glucose isomer (TGI) was determined in the SA tissue (0.59 g/Kg), followed by CR and FE cultivars with similar concentrations (*p* > 0.05), but 55% lower than SA (Table 1). The concentration of this gallotannin in the residual tissue was 155% higher than that reported for the edible part of the fruit (0.0038 g/Kg) in ‘San Andreas ‘ strawberry, and ‘Black Satin ‘ blackberry (0.12 g/Kg) (Van de Velde et al. 2019). The ellagic acid pentoside (EAP) concentration was 95 times higher in the FE cultivar (0.760 mg/Kg) (*p* < 0.05) than in CR and SA. The concentration of this phenolic compound was higher than that reported for the whole fruit, e.g., 0.1787 g/Kg for strawberry cv*.* ‘Albion’ and 0.178 g/Kg for strawberry cv. ‘Chandler’ (Ibanez et al. 2019). These results agree with the fact that strawberry fruits, and in turn their by-products, are excellent sources of ellagic acid and its derivatives for the human diet (Alfei et al. 2019). Table 2 shows a positive correlation (*p* < 0.05) of this compound with the antioxidant capacity, pointing out the contribution of ellagic acid pentoside in the *in-vitro* antiradical activity of the different tissues.

**Table 1:** Phenolic compounds content from the by-products of three strawberry cultivars

Cultivar	TGI (g/Kg R)	EAP (g/Kg R)	AGN (g/Kg R)	EA (g/Kg R)	Q3G (g/Kg R)	K3G (g/Kg R)	KP (g/Kg R)	P3G (g/Kg R)
CR	0.341 ± 0.023b	0.008 ± 0.012b	0.386 ± 0.256c	0.033 ± 0.042a	0.132 ± 0.011ab	0.072 ± 0.007a	0.010 ± 0.001a	0.027 ± 0.001b
SA	0.599 ± 0.097a	0.003 ± 2E−04b	0.894 ± 0.292b	0.015 ± 0.003a	0.075 ± 0.028b	0.112 ± 0.021a	0.005 ± 0.001b	0.069 ± 0.011a
FE	0.266 ± 0.032b	0.760 ± 0.079a	1.556 ± 0.086a	0.072 ± 0.022a	0.293 ± 0.122a	0.156 ± 0.093a	0.009 ± 7E−04a	0.019 ± 9E−04b

Different lowercase letter indicates significant differences (*p* < 0.05) by Tukey’s test among cultivars

*CR* camino real, *SA* San Andreas y, *FE* festival, *TGI* tetragalloyl-glucose isomer, *EAP* ellagic acid pentoside, *AGN* agrimoniin, *EA* free ellagic acid, *Q3G* quercetin-3-*O*-glucuronide, *K3G* kaempferol-3-*O*-glucuronide, *KP* kaempferol pentoside, *P3G* pelargonidin-3-glucoside

**Table 2:** Correlations coefficients for different variables studied

	TGI	EAP	AGN	EA	Q3G	K3G	KP	P3G	TPC_HPLC_	TPC		CAO
TGI	–											
EAP	NS	–										
AGN	NS	0.8365**	–									
EA	NS	0.7145*	NS	–								
Q3G	− 0.7236*	0.8374**	NS	0.7840*	–							
K3G	NS	NS	NS	NS	0.6853*	–						
KP	− 0.8268**	NS	NS	0.6682*	0.6742*	NS	–					
P3G	NS	NS	NS	NS	NS	NS	− 0.8655**	–				
TPC_HPLC_	NS	0.9020***	0.9790***	NS	0.7440*	0.7326*	NS	NS	–			
TPC	NS	0.9378***	0.9185***	NS	0.7394*	NS	NS	NS	0.9598***	–		
CAO	NS	0.7601*	0.8768**	NS	NS	0.7039*	NS	NS	0.9000***	0.8935**	–	

*TPC* total phenolic content, *CAO* antioxidant capacity, *TPC*_*HPLC*_ total phenolic compounds analyzed by HPLC, *TGI* tetragalloyl-glucose isomer, *EAP* ellagic acid pentoside, *AGN* agrimoniin, *EA* free ellagic acid, *Q3G* quercetin-3-*O*-glucuronide, *K3G* kaempferol-3-*O*-glucuronide, *KP* kaempferol pentoside, *P3G* pelargonidin-3-glucoside. *NS* no significative

*p ˂ 0.05, **p ˂ 0.01, ***p ˂ 0.001

Agrimoniin (AGN) (peak (3), Fig. 3) was quantified at higher concentrations in the residual tissues of FE (*p* < 0.05), being 43% and 400% higher than the concentration determined in the residues of SA and CR, respectively. This hydrolysable tannin was the main phenolic compound in the three cultivars, confirming it as a taxonomic marker of the *Rosaceae* family (Grochowski et al. 2017). The presence of agrimoniin as the main phenolic compound has already been reported in strawberry plant leaves (Karlińska et al. 2021). Agrimoniin is a hydrolysable tannin derived from hexahydroxyphenic acid. Its galloyl groups confer important biological activities, such as antidiabetic, anticancer, hepatitis B virus protector, and anti-inflammatory (Grochowski et al. 2017; Hoffmann et al. 2016; Kashchenko et al. 2017; Wang and Jin 2011). According to the results obtained, agrimoniin was the individual phenolic compound that presented the highest correlation coefficient with the antioxidant capacity (*R*^2^ 0.87, *p* < 0.01).

The contents of free ellagic acid (EA) (peak (4), Fig. 3) did not show significant differences (*p* > 0.05) among the residual tissues of the three strawberry cultivars. Moreover, the concentrations obtained (0.015–0.072 g/Kg R) were similar to those reported for other discarded parts of plants such as black and red raspberry seeds (0.067 and 0.087 g/Kg, respectively) (Landete 2011). Hydrolysable tannins and ellagic acid derivatives were the most prevalent family of compounds in the waste tissues of the three strawberry by-products cultivars, and their content changed according to the cultivar and genotype. In general, the main phenolic compounds found in strawberry fruit are anthocyanins. In the rest of the plant (stems, leaves, and flowers), hydrolysable tannins and their derivatives can represent 40–60% of the total phenolic content (Gündüz 2015). This is due to the fact that plants of the *Rosaceae* family synthesize hydrolysable tannins and their derivatives from the oxidation of gallotannins, followed by their oligomerization. The ellagitannins synthesis defends plants against herbivorous insects, since most ellagitannins can precipitate proteins in the digestive tube of these insects, altering their metabolism and avoiding the ingestion of these types of plants (Moilanen et al. 2015). Therefore, in the non-edible parts of these plants is common to find a higher content of ellagic acid compounds and their derivatives compared to the fruit. Their concentration and distribution depend significantly on the type of cultivar and stage of development of the plant (Karlińska et al. 2021). Even though non-edible fruit tissues are an excellent source of these bioactive compounds, the hydrolysable tannins are susceptible to degradation by hydrolysis, oxidation, or both. The microencapsulation of hydrolysable tannins rich-extracts, with cyclodextrin as wall material, could increase their stability and their antioxidant capacity by up to 25% by controlling the ellagic acid release (Kaderides et al. 2020). In addition, the encapsulation of hydrolysable tannins favours their intestinal absorption, generating a beneficial effect on the gut microbiota, producing metabolites of interest such as urolithins A and B (González-Barrio et al. 2010; Jacobo-Velázquez and Cisneros-Zevallos 2020), opening new research areas for this raw material.

Flavonols constituted the second most important group identified in the residual tissues of SA, CR and FE. Tissue by-products of FE and CR strawberry cultivars presented the highest concentrations of quercetin-3-*O*-glucuronide (Q3G) (peak (5), Fig. 3), with 0.293 and 0.132 g/Kg, respectively. For the CR cultivar, quercetin-3-*O*-glucuronide represented 13% of the total phenolic compounds identified, followed by the cultivar FE with 9.34%, and finally SA with 4.2%. The CR and FE cultivars had higher concentrations than those reported by Fotirić Akšić et al. (2019) for organic blueberry leaves cv. ‘Nui’ (0.1302 g/Kg), and organic strawberry leaves cv. ‘Clery’ (0.0133 g/Kg) grown in Serbia. For kaempferol-3-*O*-glucuronide (K3G) (peak (6), Fig. 3), there were no significant differences (*p* > 0.05) among the residues of the cultivars studied. Besides, K3G showed a significant correlation (*p* < 0.05) with antioxidant capacity (*R*^2^ 0.70) similar to that reported by Zielinski et al. (2016) for the phenol-rich extract of white tea (*R*^2^ 0.77). For the kaempferol pentoside (KP) (peak (7), Fig. 3), the residual tissue of CR and FE presented similar concentrations (*p* > 0.05) (0.09–0.010 g/Kg), but up to 50% higher than that found in SA. Strawberry has a significant amount of flavonols, and in some cultivars, they represent the majority of the compounds found in the fruit (Nowicka et al. 2019). Interestingly, the concentrations of kaempferol and its derivatives in the present study were higher than those reported for the fruit of 27 strawberry cultivars from Norway with an average of 0.0084 g/Kg, and 15 strawberry cultivars from Spain with a range between (0.003–0.01 g/Kg FW) (Buendía et al. 2010; Mazur et al. 2012).

Regarding pelargonidin-3-*O*-glucoside (P3G) (peak (8), Fig. 3), the SA waste tissue had the highest concentration (0.069 g/Kg), 72.4% and 60.9% higher than those found in the residues of FE and CR cultivars, respectively. Pelargonidin-3-*O*-glucoside, an anthocyanin, is responsible for the reddish colour of strawberries, and can account for 45% of the total content of the phenolic compounds in the fruit (Basu et al. 2014). The origin of this compound in the strawberry by-product is mainly due to the presence of remaining fruit tissues. As previously reported, the pelargonidin-3-*O*-glucoside was not quantified in studies carried out in strawberry by-products with less than 5% of fruit remaining, corresponding to the beginning of the harvest season (Villamil-Galindo et al. 2020). In the composition of the strawberry by-products of this work, the fruit tissue represented a low percentage; therefore, the concentration of P3G was lower than that commonly found in the strawberry fruit (0.18–0.26 g/Kg) (Aaby et al. 2012; Morales-Quintana and Ramos 2019).

The highest concentration of the sum of individual phenolic compounds was determined in the FE strawberry by-products (3.13 g/Kg R), followed by SA (1.77 g/Kg R), and finally by CR (1.00 g/Kg R), in accordance to TPC. On the other hand, the sum of these compounds presented a highly significant correlation (*R*^2^ 0.9; *p* < 0.001) with the antioxidant capacity, being higher than that reported by Aryal et al. (2019) (*R*^2^ 0.75) and Guayo and Ahia (2008) (*R*^2^ 0.88).

As mentioned previously, the variation in the profile and concentration of phenolic compounds in strawberry agro-industrial by-products is associated with genetic, geographical, or agroecological conditions under which the crop was grown (Aaby et al. 2012; Di Vittori et al. 2018; Kårlund et al. 2014). Each plant has a genetic code that is inherited from its predecessors, which added to the interaction with the environment, will present a diverse composition and distribution of nutrients and secondary metabolites, in agreement with the results obtained for the three strawberry cultivar by-products studied (CR, SA, and FE). Similarly, Aaby et al. (2012) determined the phenolic profile of 27 strawberry cultivars, reporting that genotype was the main factor affecting the variation of total phenolic content, followed by the maturity stage of the strawberry.

The biosynthesis of phenolic compounds takes place essentially due to the action of four main enzymes involved in this metabolic pathway: phenylalanine ammonium lyase (PAL), cinnamic acid 4-hydrolase (C4H), chalcone synthase (CHS), and flavonoid 3′-hydrolase (F3′H) (Francini et al. 2019). The mRNA levels of the genes encoding the expression of the different enzymes of the secondary metabolism correlate with the accumulation of the diverse phenolic compounds for each cultivar (Muñoz et al. 2011).

Ellagitannins are derived from the shikimic metabolic pathway. In this work, the waste tissues of the FE cultivar showed a higher accumulation of these hydrolysable tannins. However, since ellagitannins have the same metabolic pathway that the flavonoids (flavonols), there may be expected an inverse correlation between the content of both kinds of compounds, and endogenous factors will determine the proportion of these compounds that will be synthesized. In the case of TGI, Table 2 shows a negative correlation (*p* < 0.05) with Q3G and KP, indicating that for the inedible part of the strawberry fruit, probably the secondary metabolism of the shikimic acid follows the synthesis of the TGI compound than to the synthesis of the flavonols Q3G and KP.

Regarding extrinsic factors, Wang and Camp (2000) found that an increase in strawberry crop temperature above 30/22 °C (day/night, respectively) produced a decrease in the concentration of ellagic acid and its derivatives in leaves and fruits. Those temperatures would modulate the entire metabolic pathway flow towards flavonoid production due to its ability to absorb UV radiation, reflected by an increase in CHS enzyme activity (Kelly 2018). Consequently, the higher accumulation of ellagitannins, gallotannins, and their derivatives in the FE by-products, probably occurs, because it is an early cultivar harvested in the fall–spring seasons (lower sunlight hours), and its metabolic pathway is more productive towards hydrolysable tannins (Axel 2016; Sordo et al. 2017).

The genus *Fragaria* is characterized by its high flavonoid content and the wide variability in the concentration and disposition of these metabolites (Muñoz et al. 2011). Flavonoids are synthesized in the plant cell cytoplasm and then transported to the vacuoles. However, flavonoids are more stable at vacuole pH in their glycosylated forms (Halbwirth et al. 2006), and as determined in the present work may be degraded when exposed to an extracellular medium, lowering their recovery. A high amount of flavonoids in different parts of the strawberry plant has been associated with a decreased susceptibility to fungal attack (Mikkonen et al. 2002). In this study, FE by-products had the highest flavonoid concentrations (0.458 g/Kg), indicating a lower susceptibility to fungal attack than the other cultivars, a critical characteristic for their management (Fig. 4). As strawberry by-products are very perishable, if they are not disposed of appropriately, these metabolites of interest may be lost additionally to the environmental impact. The use of ultrasound assisted-extraction is a low-cost way to revalue this by-product. This technology is safe, reduces extraction times, and is industrially profitable and feasible (Welti-Chanes et al. 2017). Moreover, using green solvents such as water or ethanol instead of methanol to obtain food-grade extracts is an alternative for their application in the fresh-cut industry due to the extract capacity to inhibit the polyphenol-oxidase enzyme (Villamil-Galindo et al. 2020).

## Conclusions

The current study determined the composition and concentration of phenolic compounds in agro-industrial by-products from strawberry processing of ‘Festival’, ‘San Andreas’, and ‘Camino Real’ cultivars. The total phenolic content, antioxidant capacity, and phenolic profile varied significantly among cultivars. The waste tissue of the ‘Festival’ cultivar had the highest concentration of phenolic compounds, being up to 2.4 times higher than the ‘Camino Real’ cultivar, and together with the ‘San Andreas’, had the highest antioxidant capacity. Eight phenolic compounds were identified and quantified from strawberry by-products. The hydrolysable tannins were the main compounds determined in the three cultivars studied, representing more than 80% of the total phenolic compounds for the ‘Festival’ and ‘San Andreas’ strawberry cultivar by-products. Agrimoniin was the principal hydrolysable tannin determined in the strawberry by-products, with concentrations between 0.38 and 1.56 g/Kg, having a highly significant correlation with the antioxidant capacity of the three cultivars. The ‘Camino Real’ cultivar had the highest concentration of flavonols compounds, especially kaempferol-3-*O*-glucuronide, which significantly correlated with the antioxidant capacity.

Therefore, the changes in phenolic compounds and their antioxidant capacity confirm the impact of the genotype in the bioactive compound concentrations. This knowledge allows adequate recovery of the metabolites of interest according to the cultivar, as well as its appropriate management, considering the susceptibility to fungal attack, hydrolysis, and oxidation, according to the phenolic compound profile. As a result, these strawberry by-products can be revalorised as a low-cost source of antioxidant compounds with potential application for the nutraceutical industry.

## Data Availability

All data generated or analyzed during this study are included in this article.

## Abbreviations

FE: Festival cultivar
SA: San Andreas cultivar
CR: Camino Real cultivar
TPC: Total phenolic content
GAE: Gallic acid equivalents
TE: Trolox equivalent
R: Strawberry by-products
FW: Fresh weigh
TGI: Tetragalloyl-glucose isomer
EAP: Ellagic acid pentoside
AGN: Agrimoniin
EA: Ellagic acid
Q3G: Quercetin-3-*o*-glucuronide
K3G: Kaempferol-3-*o*-glucuronide
KP: Kaempferol pentoside
P3G: Pelargonidin-3-*o*-glucoside
PAL: Phenylalanine ammonium lyasa
(C4H): Cinnamic acid 4-hydrolase
CHS: Chalcone synthase
F3´H: Flavonoid-3′-hydrolase
